# Long-Term Effects of Altered Photoperiod During Pregnancy on Liver Gene Expression of the Progeny

**DOI:** 10.3389/fphys.2019.01377

**Published:** 2019-11-22

**Authors:** Pamela Carmona, Bárbara Pérez, Carlos Trujillo, Gabriel Espinosa, Fernando Miranda, Natalia Mendez, Claudia Torres-Farfan, Hans G. Richter, Karina Vergara, Priscilla Brebi, José Sarmiento

**Affiliations:** ^1^Laboratorio de Cronoinmunología, Instituto de Fisiología, Facultad de Medicina, Universidad Austral de Chile, Valdivia, Chile; ^2^Programa de Doctorado en Ciencias Médicas, Universidad de La Frontera, Temuco, Chile; ^3^Programa de Doctorado en Ciencias Veterinarias, Universidad Austral de Chile, Valdivia, Chile; ^4^Laboratorio de Cronobiología del Desarrollo, Instituto de Anatomía, Histología y Patología, Facultad de Medicina, Universidad Austral de Chile, Valdivia, Chile; ^5^Laboratorio de Patología Molecular, Departamento de Patología, Facultad de Medicina, Universidad de La Frontera, Temuco, Chile

**Keywords:** cardiovascular disease, fibrinolytic system, *Pai-1*, clock genes, gestational chronodisruption, DOHaD

## Abstract

Experimental and epidemiological studies have revealed a relationship between an adverse intrauterine environment and chronic non-communicable disease (NCD) like cardiovascular disease (CVD) in adulthood. An important risk factor for CVD is the deregulation of the fibrinolytic system particularly high levels of expression of plasminogen activator inhibitor 1 (*Pai-1*). Chronic exposure to altered photoperiod disrupts the circadian organization of physiology in the pregnant female, known as gestational chronodisruption, and cause long-term effects on the adult offspring’s circadian physiology. The *Pai-1* expression is regulated by the molecular components of the circadian system, termed clock genes. The present study aimed to evaluate the long-term effects of chronic photoperiod shifts (CPS) during pregnancy on the expression of the clock genes and the fibrinolytic system in the liver of adult male offspring. Our results using an animal model demonstrated statistically significant differences at the transcriptional level in males gestated under CPS. At 90 days of postnatal age, the liver transcript levels of the clock gene *Bmal1* were downregulated, whereas *Ror*α, *Ror*γ, *Nfil3*, and *Pai-1* were upregulated. Our data indicate that CPS during pregnancy affects gene expression in the liver of male adult progeny, showing that alteration of the photoperiod in the mother’s environment leads to persistent effects in the offspring. In conclusion, these results reveal for the first time the long-term effects of gestational chronodisruption on the transcriptional activity of one well-established risk factor associated with CVD in the adult male offspring.

## Introduction

The environment during early life influences the risk of developing pathophysiological processes later; the field recognized as the developmental origins of health and disease (DOHaD) (Calkins et al., 2011; Capra et al., 2013; Hanson and Gluckman, 2014). In particular shows an association with NCDs (Fowden et al., 2006; Marsh et al., 2011) as CVD (Thompson and Trask, 2016).

In modern society, exposure to environmental light at night (e.g., chronic shift work, work at night), disarranging the internal biological clock; thus producing a significant disturbance of the circadian organization of physiology known as chronodisruption (Erren and Reiter, 2009). Circadian rhythms are intrinsic biological oscillations with a 24-h period driven by the circadian timing system, coordinating physiology and behavior with the daily light/dark cycle (Mazzoccoli et al., 2012; Partch et al., 2014; Tarquini and Mazzoccoli, 2017). This system is organized by the central clock, located in the suprachiasmatic nucleus; which is entrained by the light/dark cycle as a dominant signal, in addition to several peripheral clocks located throughout the body. At the cell level, circadian rhythmicity relies on clock gene expression in central and accessory interlocking transcription/translation feedback loops (TTFL) (Mohawk et al., 2013; Curtis et al., 2014; Takahashi, 2016). In turn, these core clock genes promote the expression of downstream genes (CCGs) (Albrecht, 2012; Liu and Chu, 2013). Significant changes in the expression of clock genes can affect physiological processes controlled by the biological clock and have been associated with the development of NCDs (Plano et al., 2017; Touitou et al., 2017).

The misalignment of the maternal circadian system (gestational chronodisruption) impacts fetal health (Serón-Ferré et al., 2012, 2013). This field is of great interest because of the potential long-term effects on the adult offspring’s health and disease status (Amaral et al., 2014; Varcoe et al., 2017; Richter et al., 2018). The available evidence has demonstrated different consequences of chronodisruption on maternal physiology (Gatford et al., 2019). In animal model, the maternal exposure to CPS disrupted the biological clocks in the pregnant female, altering physiological parameters throughout gestation such as the circadian profile of plasma hormones, changes in the liver metabolic gene expression and alterations in the clock gene expression profile (Varcoe et al., 2013; Mendez et al., 2016). Meanwhile, in the adult offspring gestational chronodisruption induced effects such as hyperleptinemia, hyperinsulinemia, impaired glucose tolerance (Varcoe et al., 2011); alterations in the plasma circadian profile of melatonin and corticosterone (Mendez et al., 2016); as well as alteration of adrenal endocrine messengers. In fact, there is strong evidence suggesting that the adrenal gland loses the ability to respond to ACTH (Mendez et al., 2016; Salazar et al., 2018). Given that the endocrine adrenal outputs play a key role in the development and entrainment of the fetal clock in the suprachiasmatic nucleus (Čečmanová et al., 2019), coordinating metabolic responses and acting as time-giving signals to other peripheral circadian oscillators such as the liver (Pezük et al., 2012); long-term alterations of adrenal function can lead to multiple pathophysiological processes.

The liver is a well-described peripheral clock and as such, its physiology is controlled by circadian rhythms, the clock regulates the transcription of CCGs that participate in a wide array of the physiological process in the liver (Reinke and Asher, 2016; Tahara and Shibata, 2016; Zwighaft et al., 2016) and the evidence supports that synchronized liver clockwork machinery develops gradually during ontogenesis (Sumová et al., 2008). On the other hand, the liver is the major site of *Pai-1* synthesis, being regulated transcriptionally by endocrine signals of the adrenal gland, which in turn strongly responds to light input (Dimova and Kietzmann, 2008; Aoshima et al., 2014). Also, *Pai-1* is a CCG (Haus, 2007) and its expression is upregulated through binding of the CLOCK: BMAL heterodimer to E-box sites of the *Pai-1* gene promoter region (Maemura et al., 2000; Schoenhard et al., 2003; Ohkura et al., 2006). Also, the transcription of *Pai-1* is promoted by RORα and repressed by REV-ERBα acting on RORE sites (Wang et al., 2006), all of them important members of the clock molecular machinery. Of note, epidemiological studies identify PAI-1 as a risk factor for CVD (Tofler et al., 2016; Jung et al., 2018).

Our hypothesis is that gestational chronodisruption promotes changes in the adult offspring, specifically, alterations of the regulation of molecular machinery of the liver clock genes; which in turn regulate the transcriptional pattern of the *Pai-1* in the liver. To test our hypothesis, we used a rat model of gestational chronodisruption. Our specific aims were to investigate the impact of prenatal CPS in the liver of adult male progeny on (1) clock gene transcription patterns; and (2) the fibrinolytic system, particularly in the *Pai-1* transcriptional levels.

## Materials and Methods

### Animals

Animal handling and care followed the Guide for the Care and Use of Laboratory Animals of the Institute for Laboratory Animal Research of the National Research Council. The protocols were approved by the Bioethics Commission of the Universidad Austral de Chile (CBA number 267/2016).

**FIGURE 1:**
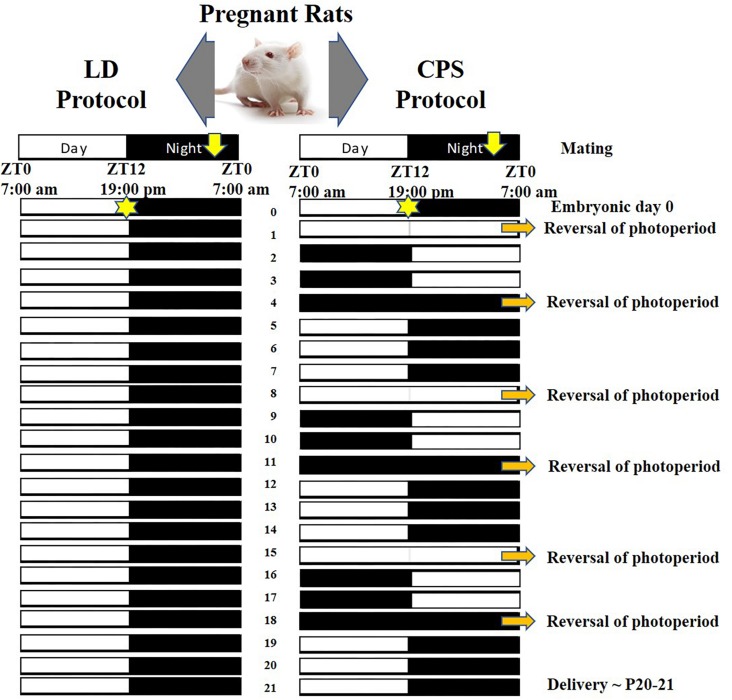
Light dark (LD) and CPS protocol scheme during pregnancy. **(Left)** LD control or standard protocol; 12 h light, 12 h dark cycle [lights on at 7:00 AM (ZT0), lights off at 7:00 PM (ZT12)]. **(Right)** CPS protocol; lighting schedule manipulation every 3–4 days, some days of constant light or constant darkness are required to reversing the photoperiod completely (orange narrow) (Mendez et al., 2016).

The animals were maintained in a control (standard) photoperiod [12 h light, 12 h dark cycle; lights on at 7:00 AM (ZT0), lights off at 7:00 PM (ZT12)]; ∼400 lux at the head level, temperature (18–20°C), humidity (∼48%), food and water were available *ad libitum* (Mendez et al., 2016; Salazar et al., 2018). Sprague Dawley rats (obtained from Charles River Laboratories International Inc.) were mated and raised in our animal facility. Timed-pregnant females were used in the study, and the day in which spermatozoa were observed in the smear of the vaginal contents was considered embryonic day 0 (E0). The pregnant females were separated by weight pairing and allocated to the following two photoperiods: light/dark (LD; control photoperiod) and CPS, using the same protocol reported by Mendez et al. (2016). Briefly, pregnant females were exposed to lighting schedule manipulation every 3–4 days, reversing the photoperiod completely, during 18 days of pregnancy (Figure 1). At 18 days of gestation, the mothers returned to a control 24-h photoperiod (12:12, lights on at ZT0) and continued in this photoperiod thereafter.

### Effects of Gestational Chronodisruption on Daily Rhythms and mRNA Expression in Adult Offspring

After birth, both dams and pups from each pregnancy condition (LD; *n* = 12 and CPS; *n* = 6 mothers) were kept under control photoperiod and litters were weighed at postnatal age 1 day (P1) and homogenized to 10 individuals (five males and females), in order to avoid variations in weight gain. Pups were weaned at 21 days old, with the males being raised in the control photoperiod (LD) to be studied at P90 (LD and CPS, *n* = 30 each group).

Body weight was measured from 30 days old, every 7 days. Males from each pregnancy condition were euthanized at P90 every 4 h for six samplings over 20 h, in LD and CPS (*n* = 5/each time point), starting at ZT1 and ending at ZT21. To avoid litter effects, each clock time point contained animals from different mothers; thus, no siblings were used at the same time point. Briefly, male rats were deeply anesthetized (isoflurane 3.5%, Baxter Laboratories), a blood sample was collected from the vena cava, and then an overdose of T61 (0.5 ml/kg body weight; Merck Animal Health, Intervet Canada Corp., Kirkland, QC, Canada) was delivered at the same site. Organs were collected, weighed and stored in RNA stabilization solution (RNAlater^TM^ Invitrogen) at 4°C for 24 h and subsequently at −20°C in our tissue bank.

### RNA Extraction and Quantitative Real-Time PCR (RT-PCR) Analysis of the Liver

Relative quantification by RT-PCR was used to evaluate the mRNA expression of clock genes and fibrinolytic system genes at P90. Total RNA was extracted using the SV Total RNA Isolation System (Promega) according to the manufacturer’s instructions. The amount of 2.0 μg of total RNA was reverse transcribed using random primers (Promega) and MLT-V reverse transcriptase (Promega). RT-PCR was performed using primers described in Supplementary Table 1 and KAPA SYBR FAST quantitative PCR master mix (Kapa Biosystems, Inc.). Quantitative PCR was carried out in a Rotor-Gene Q real-time platform (QIAGEN). Serial dilutions of cDNA were amplified by real-time PCR using specific primers for target and reference gene and determining template dilution for the sample’s measurements. A melting curve analysis was performed on each sample after the final cycle to ensure that a single product was obtained. Relative amounts of all mRNAs were calculated by the comparative ΔΔ cycle threshold method using the equation 2^–ΔΔCt^ to linearize the data and then perform the statistical analysis (Livak and Schmittgen, 2001) and normalized to the corresponding 18S-rRNA housekeeping level. For the analysis of daily rhythms we follow the Guidelines for Genome-Scale Analysis of Biological Rhythms (Hughes et al., 2017) and three independent methods were used; single cosinor (Refinetti et al., 2007), JTK_Cycle (Hughes et al., 2010), and RAIN’s longitudinal mode (Thaben and Westermark, 2014).

### Statistical Analysis

Statistical analyses were performed using the IBM SPSS software 20.0. The normality of data distribution was determined by the Shapiro Wilk test, and homogeneity of variances was analyzed by Levene’s test. The body weight was analyzed by a two-way ANOVA test for repeated measures in one of the factors, with the Bonferroni adjustment and data were expressed as mean ± SEM. Transcript levels between the two groups were analyzed by the Mann–Whitney *U* test and Student’s *t*-test.

## Results

### Impact of Gestational CPS on Liver Clock Genes Expression in Adult Male Offspring

**FIGURE 2:**
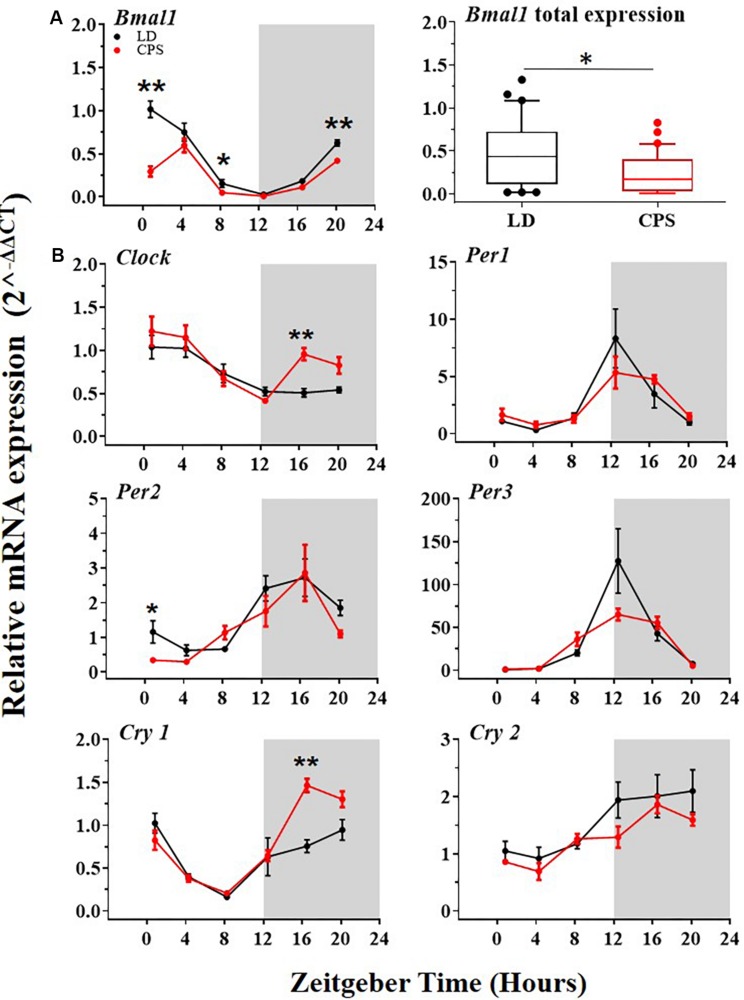
The transcription level of clock genes of the central loop in the liver from 90-day-old male rats by RT-PCR. [**A**(left),**B**] Detection of daily rhythm. Black symbols represent males gestated under control conditions (LD, black dots), and red symbols indicate males gestated in CPS (CPS, red dots). Males from each pregnancy condition (LD: *n* = 12 and CPS: *n* = 6 mothers) in LD and CPS offspring (*n* = 5/each time point). Time is expressed as zeitgeber time (ZT), with ZT0 as time lighting onset and ZT12 as lighting end; the gray bar indicates lights off. The RAIN’s longitudinal mode, JTK_Cycle and the single cosinor method were used to determine daily rhythm (*p* < 0.05), solid black and red lines represent the detection of a 24-h daily rhythm for the three methods. (**A**,left) Data for *Bmal1* are shown. ^*^p < 0.05 indicate differences between LD and CPS for time point (Mann–Whitney *U* test). (**A**,right) Daily total expression. Data for *Bmal1*, minimum, first quartile, median, third quartile, and maximum were for LD offspring: *Bmal1*: 0.02, 0.11, 0.44, 0.73, and 1.33; and for CPS offspring: 0.01, 0.03, 0.17, 0.40, and 0.83. ^*^p < 0.05. Different from LD (Mann–Whitney *U* test). **(B)** Data for other clock genes are shown ^*^p < 0.05, ^∗∗^*p* < 0.01 Different from LD for time point (Mann–Whitney *U* test).

The body weight data showed that gestational CPS in both, newborn male (P1) and adult male (P90) offspring was statistically greater than in LD progeny (P1: 7.1 ± 0.09 g CPS vs. 6.78 ± 0.07 g LD, unpaired *t*-test *p* = 0.01; P90: 499.2 ± 5.9 g CPS vs. 476.2 ± 7.9 g LD, ANOVA two way test for repeated measures with Bonferroni adjustment *p* = 0.032). We found no differences in the weight of female offspring at P1, size of the litters, and weight of the liver, lung, thymus, and spleen at P90 between LD and CPS (Supplementary Table 2 and Supplementary Figure 1). Next, we evaluated the effects of gestational CPS on the liver clock genes expression at the transcript level in the male offspring at P90. Our results showed daily rhythm expression of *Bmal1* in the offspring from both conditions (Figure 2A, left and Supplementary Tables 3–5), but with significantly reduced mRNA expression in the progeny gestated under CPS conditions (Figure 2A, right). Interestingly we found that the daily peak of *Clock* and *Nfil3* gene at transcript level was changed by 4 h (ZT 3.7, 4.0, and 1 to 0.4, 0.0, 21 for Cosinor, JKT_Cycle and RAN, respectively) between CPS and LD gestated adult progeny (Supplementary Tables 3–5). *Bmal1* and *Clock* are an important positive component of the central loop and promotes the expression of other clock genes. For this reason, we determined the transcript level of other genes of the central loop of the molecular clock (*Clock*, *Per1*, *Per2*, *Per3*, *Cry1*, and *Cry2*). The analysis of results showed that the daily peak of expression in the control condition (LD) of all these genes was in agreement with the reported ZT in other studies on the adult rat (Sládek et al., 2007; Figure 2B, black dots and Supplementary Tables 3–5). More importantly, the genes positively regulated by *Bmal1* of the central loop evaluated here showed a similar daily rhythm of expression in both progenies with no remarkable effect on the daily rhythm pattern (Figure 2B and Supplementary Tables 3–5) or total expression (data not shown) at the mRNA level in LD and CPS male offspring.

**FIGURE 3:**
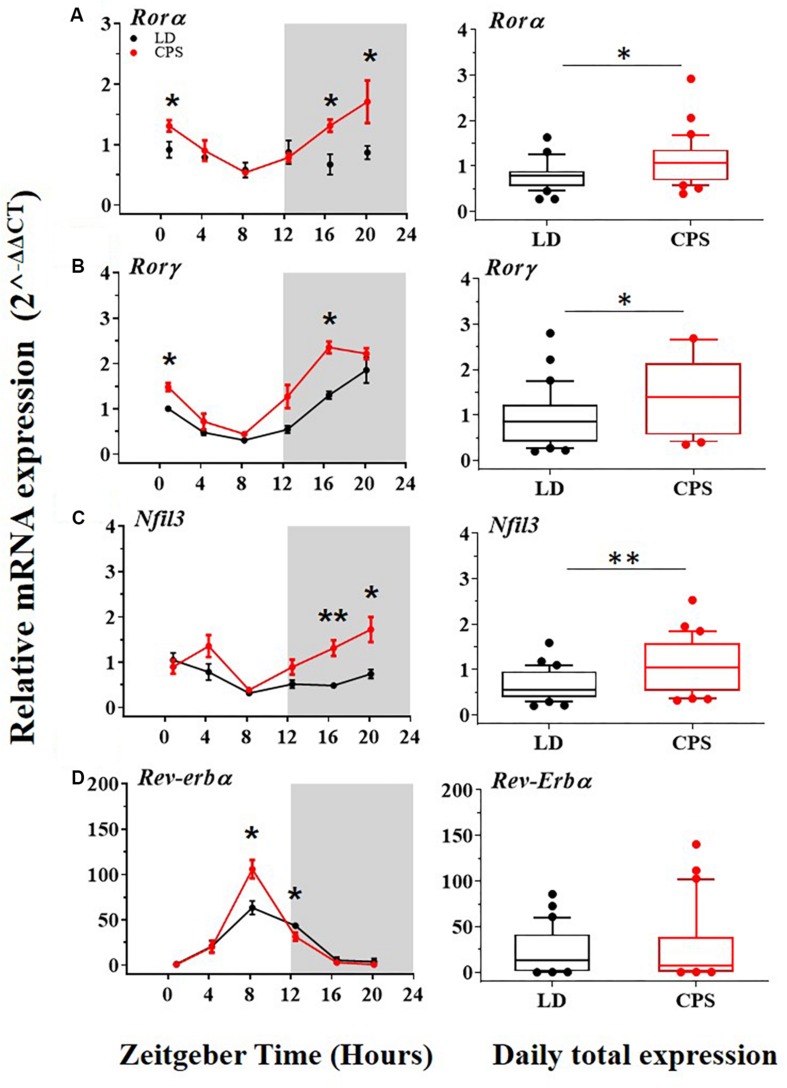
The transcription level of clock genes of the accessory loop in the liver from 90-day-old male rats by RT-PCR. (**A–D**,left) Detection of daily rhythm. Black symbols represent males gestated under control conditions (LD, black dots), and red symbols indicate males gestated in CPS (CPS, red dots). Males from each pregnancy condition (LD: *n* = 12 and CPS: *n* = 6 mothers) in LD and CPS offspring (*n* = 5/each time point). Time is expressed as zeitgeber time (ZT), with ZT0 as time lighting onset and ZT12 as lighting end; the gray bar indicates lights off. The RAIN’s longitudinal mode, JTK_Cycle and the single cosinor method were used to determine daily rhythm (*p* < 0.05), solid black and red lines represent the detection of a 24-h daily rhythm for the three methods. ^*^p < 0.05, ^∗∗^*p* < 0.01. Different from LD for that time point (Mann–Whitney *U* test). (**A–D**,right) Daily total expression. Data and median with interquartile range are shown, in LD and CPS (*n* = 30) offspring. ^*^p < 0.05, ^∗∗^*p* < 0.01. Different from LD (Mann–Whitney *U* test). Minimum, first quartile, median, third quartile, and maximum were for LD offspring: *Ror*α:0.27, 0.55, 0.78, 0.86, and 1.63; *Ror*γ: 0.2, 0.41, 0.86, 1.23, and 2.80; *Nfil3*: 0.2, 0.38, 0.55, 0.96, and 1.59; and for CPS offspring: *Ror*α: 0.39, 0.68, 1.07, 1.44, and 2.92; *Ror*γ: 0.35, 0.58, 1.42, 2.15, and 5.6; *Nfil3*: 0.32, 0.53, 1.07, 1.64, and 2.53.

Interlocked with the central loop there is an additional well-established secondary or accessory loop that involves REV-ERB and RORs transcription factors which influence negatively and positively, respectively on *Bmal1* transcription by binding to its promoter site (RORE site), and also regulate the expression of the Nuclear Factor Interleukin 3 gene (Nfil3). Remarkable differences in the daily expression were found in two clockwork components of the accessory loop. The *Ror*α and *Ror*γ components of this loop were significantly increased at the mRNA level in the CPS male offspring (Figures 3A,B). Our results showed that the transcriptional level of *Nfil3* was increased in males gestated under CPS conditions (Figure 3C), with a clear acrophase in the active phase (dark phase) of the daily expression (Figure 3C, red dots and Supplementary Tables 3–5). Interestingly, *Ror*α displayed a pattern of daily rhythm only in adult males gestated in CPS with an acrophase at active phase (Figure 3A, red dots and Supplementary Tables 3–5). Differences at individuals time points but not in the daily total expression were found in the main repressor component of the accessory loop *Rev-Erb*α between CPS and LD male progeny (Figure 3D, right and left, respectively).

### Impact of Gestational CPS on the Expression of the Fibrinolytic System in the Adult Offspring

**FIGURE 4:**
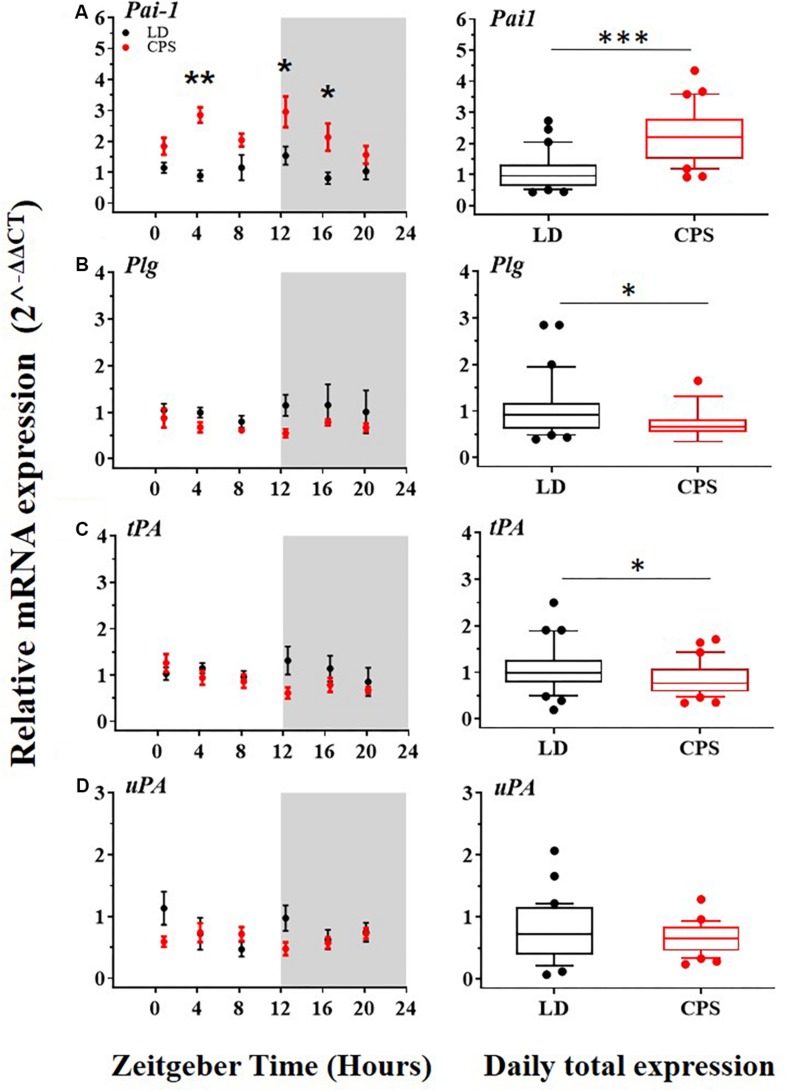
The transcription level of *Pai-1*, *Plg*, *tPA*, and *uPA* in the liver from 90-day-old male rats by RT-PCR. (**A–D**,left) Detection of daily rhythm. Black symbols represent males gestated under control conditions (LD, black dots) and red symbols indicate males gestated in CPS (CPS, red dots). Males from each pregnancy condition (LD: *n* = 12 and CPS: *n* = 6 mothers), in LD and CPS offspring (*n* = 5/each time point). Time is expressed as zeitgeber time (ZT), with ZT0 as time lighting onset and ZT12 as lighting end; the gray bar indicates lights off. The RAIN’s longitudinal mode, JTK_Cycle and the single cosinor method were used to determine daily rhythm (*p* < 0.05), solid black and red lines represent the detection of a 24-h daily rhythm for the three methods. (**A–D**,right) Daily total expression. Data and median with interquartile range are shown in LD and CPS (*n* = 30) offspring. ^*^p < 0.05, ^∗∗^*p* < 0.01, ^∗∗∗^*p* < 0.001. Different from LD (Mann–Whitney *U* test). Minimum, first quartile, median, third quartile, and maximum for LD offspring: *Pai-1*: 0.44, 0.62, 0.96, 1.33, and 2.73; *Plg*: 0.39, 0.61, 0.92, 1.15, and 2.85; *tPA*: 0.19, 0.78, 0.99, 1.27, and 2.5 and for CPS offspring: *Pai-1*: 0.92, 1.5, 2.27, 3.01, and 7.76; *Plg*: 0.34, 0.55, 0.66, 0.81, and 1.65; *tPA*: 0.34, 0.58, 0.76, 1.09, and 1.7.

Alterations of fibrinolytic activity mediated by deregulation in the expression of its components have been associated with a risk factor for CVD (Mavri et al., 2004; Oishi, 2009). Also, in the liver, the expression of important components of the fibrinolytic system is controlled by the circadian system. Our results showed that males gestated in CPS displayed significant differences in the mRNA expression level in important components of the fibrinolytic system relative to the LD group. More specifically, the main inhibitor of this system, *Pai-1*, was increased (Figure 4A, right); in contrast, the precursor of plasmin, *Plg* and *tPA*, were reduced (Figures 4B,C, right). On the other hand, *uPA* did not show significant differences between the two progenies (Figure 4D, right). Regarding daily oscillations of mRNA components of the fibrinolytic system evaluated here, we only detected a rhythm in tPA but only by RAIN method in CPS but not in LD adult male progeny (Figures 4A–D left, and Supplementary Tables 3–5).

At the protein level, the results obtained for daily plasma concentration of PAI-1 did not show significant differences between CPS and LD male offspring (Supplementary Figure 2 and Supplementary Table 6). However, daily rhythms were found for PAI-1 plasma protein concentration in both CPS and LD progeny; displaying a daily peak expression of the protein in the active phase (dark) of the circadian cycle (Ohkura et al., 2006). Notably, at P90 of development the amplitude of the oscillation of PAI-1 plasma concentration was increased in adult males which had been gestated under CPS (A = 198.8 (LD) and 387.9 (CPS); AMP = 96.0 (LD) and 206.0 (CPS) determined by Cosinor and JTK_Cycle, respectively) relative to LD adult offspring (Supplementary Table 6). Finally, we found a circadian rhythm in the *ex vivo* coagulation time in both CPS and LD progeny (Supplementary Figure 3 and Supplementary Tables 7–9). Remarkably, at the postnatal age of 180 days, in CPS gestated male the time required to *ex vivo* coagulation was greater than LD. This difference showed seems to be age-dependent because it was not observed at P60 or P120 (Supplementary Figures 3A,B). These results point to a putative mayor propensity to a coagulation/fibrinolytic system imbalance in CPS than LD during the aging process.

## Discussion

Modern lifestyles are strongly correlated with misalignment of biological clocks. In this context, circadian disruption act as a sustained environmental factor that leads to conflicts between endogenous biologic clock cycles and the environment (Bass and Lazar, 2016). In the present study, gene expression patterns in the peripheral liver clock and fibrinolytic system were assessed to determine the long-term effects of gestational chronic photoperiod shifting (mimicking repeated night shift work schedules in pregnant women) on the adult offspring.

Hormonal disturbances have also been linked to altered photoperiods. Impaired secretion of corticosterone, aldosterone, and the loss of response to ACTH of the adrenal gland have been observed in progeny gestated under CPS (Mendez et al., 2016; Salazar et al., 2018). Adrenal function is directly regulated by the photoperiod as it is strictly controlled by the master clock residing in the suprachiasmatic nucleus (Aoshima et al., 2014; Plano et al., 2017). Moreover, the adrenal gland is an important oscillator from fetal to postnatal period of life (Torres-Farfan et al., 2011; Roa et al., 2017; Salazar et al., 2018) that synchronizes the rhythmic signaling of glucocorticoids and catecholamines to peripheral clocks such as the liver (Kalsbeek et al., 2012; Pezük et al., 2012). In fact, a significant desynchronization is observed in the liver of adult rats subjected to adrenalectomy (Pezük et al., 2012), strongly suggesting that the adrenal peripheral oscillator plays a crucial role in synchronizing the circadian rhythm of the liver. Previous findings in adult rats gestated under CPS indicate significant desynchronization of daily rhythms of plasma corticosterone, whereas the daily pattern of plasma ACTH was similar in both CPS and control offspring; however, corticosterone response to ACTH was lost in CPS adrenals (Mendez et al., 2016; Salazar et al., 2018). These lines of evidence could be associated with the alteration of the hepatic circadian clock. In the liver, our results demonstrated that transcript levels of *Bmal1* and the phase of the daily peak expression of *Clock* and *Nfil3* were significantly affected in adult males gestated in CPS. Interestingly, gestational CPS disrupted daily rhythms in the liver of these clock-genes even after 3 months of exposure to LD photoperiod during the postnatal developing (P90). These results reveal a long-term effect on the expression of the clock genes that changes the phenotype displayed at the adult stage under LD photoperiod in a male gestated in CPS protocol. Importantly, *Bmal1* plays a key role in the regulation of the hemostatic function of the liver and also in the progression of the prothrombotic state in aging (Hemmeryckx et al., 2011, 2019).

Alterations of the molecular clock at the accessory loop (RORE site) in the male adult progeny were also evidenced. Specifically, transcript levels of the clock genes *Ror*α and *Ror*γ (*Ror*α/γ) were increased in adult CPS males. The expression of *Ror*α/γ clock genes has been described to be positively controlled by the BMAL/CLOCK heterodimer. However, as it was described before, the transcript levels of *Bmal1* were downregulated in adult males gestated under CPS relative to LD conditions. That can be explained by the fact that posttranslational modifications of the BMAL/CLOCK heterodimer have been shown play a key role in terms to modify its activity independently of mRNA level regulation (Hirayama et al., 2007; Bellet and Sassone-Corsi, 2010; Preußner and Heyd, 2016). Additionally, it has been demonstrated that *Ror*α/γ expression is controlled by other circadian signals via cAMP response elements (CREs) (O’Neill et al., 2008) that could increase its role under the effects of gestational CPS. In order to determine if increased transcript levels of *Ror*α/γ may have functional consequences, we evaluated transcript levels of *Nfil3* in the liver, because its expression is principally regulated by *Ror*γ (Ueda et al., 2005; Takeda et al., 2012). The transcription level of *Nfil3* was significantly increased in adult males gestated in CPS. Also, the phase (ZT) of the daily peak expression of *Nfil3* was significantly affected (3.5 h) in males gestated in CPS (Supplementary Tables 3–5). This finding emphasizes that the effects on the accessory loop of the molecular clock could deregulate CCGs and therefore alter physiological functions. Moreover, endocrine signal as insulin is also important in the regulation of the expression of *Nfil3* (Keniry et al., 2014) and as previously mentioned, hyperinsulinemia has been reported in adult offspring gestated in CPS (Varcoe et al., 2011).

A reduced fibrinolytic activity due to an increase in the expression of PAI-1 is a characteristic risk factor for CVD (Mavri et al., 2004; Oishi, 2009). Liver physiology is heavily involved in the regulation of fibrinolytic activity since many of its components, like plasminogen (Cesarman-Maus and Hajjar, 2005; Leebeek and Rijken, 2015) and PAI-1 (Oishi, 2009; Declerck and Gils, 2013) are mainly synthesized by this organ. In adult mice, it has been reported that chronic alteration of the photoperiod was associated with the deregulation of the *Pai-1* expression in the liver (Oishi and Ohkura, 2013). On the other hand, previous evidence indicates that clock genes regulate the expression of PAI-1 (Schoenhard et al., 2003; Ohkura et al., 2006; Wang et al., 2006). For instance, a mouse model deficient in *Bmal1* (*Bmal1^–/–^*) displayed elevated plasma levels of PAI-1, which were associated with a prothrombotic phenotype (Hemmeryckx et al., 2011; Somanath et al., 2011). Regarding gestational chronodisruption, our results showed increased levels of *Pai-1* in the liver of male adult offspring gestated under CPS. This observation is relevant because we previously showed a significant increase in blood pressure in CPS males at P90 (Mendez et al., 2016). Both, increased levels of PAI-1 associated with clock genes deregulation here reported and high pressure described previously are recognized like a CVD risk factors. Some factors that induce *Pai-1* gene expression are insulin, glucocorticoids (Irigoyen et al., 1999; Mavri et al., 2004; Dimova and Kietzmann, 2008). Interestingly, factors as hyperinsulinemia and alteration of corticosterone circadian rhythm also described in this animal model (Varcoe et al., 2011; Mendez et al., 2016) have been shown to induce greater expression of *Pai-1* in the liver. On the other hand, our data showed a decreased expression of *Bmal1* clock gene in the males gestated in CPS. Previous evidence supports the idea that reduced expression of *Bmal1* in the liver results in increased expression levels of *Pai-1*. In particular, the upregulation of PAI-1 is associated with an increase in thrombosis propensity during the aging process (Hemmeryckx et al., 2011, 2019; Somanath et al., 2011). In addition, REV-ERBα is a negative regulator of *Pai-1* (Wang et al., 2006) expression by a mechanism involving its competition with RORα, a positive regulator that our results showed that is increased at transcript level and also rhythmic with a phase (ZT) of the daily peak expression in the active phase (dark) in CPS adult progeny. We did not observe differences in *Rev-Erb*α daily total expression between adult males gestated under CPS and LD conditions, suggesting an inclination to induce *Pai-1* expression by RORα rather than repression by REV-ERBα.

The appearance of a marked daily oscillation at the transcript level of the clock gene *Ror*α in adult CPS but not in LD offspring was another interesting finding. The induction of rhythmic expression of genes that are not oscillatory could be mediated by epigenetic mechanisms, which are involved in the regulation of the transcriptional machinery and reveal that expression of genes that are not rhythmic could be induced (Masri et al., 2014). The induction of the rhythmic expression pattern in *Ror*α in CPS adult males suggests that epigenetic mechanisms might play a role in the long-term effects observed.

At the protein level, results obtained for daily plasma concentration of PAI-1 did not show significant differences between CPS and LD male offspring. However, daily rhythms were found for PAI-1 plasma protein; displaying a daily peak expression of the protein in the active phase (dark) of the daily cycle that are in agreement with data previously reported in rodents for LD condition (Ohkura et al., 2006). Notably, at P90 of development, the amplitude of the oscillation of plasma concentration in the active phase was increased in adult males which had been gestated under CPS relative to LD offspring (Supplementary Table 6).

It has been described that older humans are more susceptible to thrombosis under septic conditions (Balleisen et al., 1985; Aillaud et al., 1986). In addition, murine models have been demonstrated that the aging process increases the endotoxin-induced thrombosis by a mechanism that involves increased expression of PAI-1 protein in the plasma and at mRNA level in the liver. This tendency is linked to an enhanced inflammatory response in aged mice (Yamamoto et al., 2002). Connected with this previous literature we found a circadian rhythm in the *ex vivo* coagulation time that was increased at postnatal age of 180 days in CPS gestated male in a process that is age-dependent because this difference was not observed at P60 or P120.

## Data Availability Statement

The datasets generated for this study are available on request to the corresponding author.

## Ethics Statement

The animal study was reviewed and approved by Bioethics Commission of the Universidad Austral de Chile (CBA number 267/2016).

## Author Contributions

PC and JS contributed to the conception, design, and drafting of the work. PC wrote the first draft of the manuscript. PC, BP, CT, NM, GE, and KV worked on animal handling and acquisition of data. PC, JS, and FM analyzed and interpreted the data. CT-F, HR, and PB critically reviewed the manuscript for important intellectual content. All authors contributed to the manuscript revision, and read and approved the submitted version.

## Conflict of Interest

The authors declare that the research was conducted in the absence of any commercial or financial relationships that could be construed as a potential conflict of interest.

## Abbreviations

CCG: clock-controlled gene
CPS: chronic photoperiod shifts
CVD: cardiovascular disease
E18: embryonic day 18
LD: light dark
NCD: non-communicable disease
*Pai-1*: plasminogen activator inhibitor-1
*Plg*: plasminogen
P1: postnatal day 1
P60: postnatal day 60
P90: postnatal day 90
P120: postnatal day 120
ROR: retinoid-related orphan receptor
RORE: ROR-response elements
tPA: tissue Plasminogen Activator
uPA: urokinase Plasminogen Activator
ZT: zeitgeber time.
