# Supplementary material for: Long-Term Effects of Altered Photoperiod During Pregnancy on Liver Gene Expression of the Progeny
Source: Front Physiol. 2019 Nov 22;10:1377. doi: 10.3389/fphys.2019.01377 (PMC6883370; doi:10.3389/fphys.2019.01377)
Supplement: Supplementary file 1 [file Data_Sheet_1.PDF]

SUPPLEMENTAL MATERIAL

SUPPLEMENTAL TABLES

Table 1. RT-PCR primers for liver gene expression studies.

		Primers		
**Gene**	**Access number**	**Forward**	**Reverse**	**Amplicon size (bp)**
18S	NR_046237.1	GTAACCCGTTGAACCCCATT	CCATCCAATCGGTAGTAGCG	151
Bmal1	NM_024362	CCGATGACGAACTGAAACACCT	TGCAGTGTCCGAGGAAGATAGC	215
Clock	NM_021856	TCTCTTCCAAACCAGACGCC	TGCGGCATACTGGATGGAAT	110
Per 1	XM_340822	TCTGGTTCGGGATCCACGAA	GAAGAGTCGATGCTGCCAAAG	101
Per 2	NM_031678	CACCCTGAAAAGAAAGTGCGA	CAACGCCAAGGAGCTCAAGT	148
Per 3	NM_023978	ATAGAACGGACGCCAGAGTGT	CGCTCCATGCTGTGAAGTTT	104
Cry1	NM_198750	AAGTCATCGTGCGCATTTC	TCATCATGGTCGTCGGAC	196
Cry2	NM_133405	GGATAAGCACTTGGAACGGAA	ACAAGTCCCACAGGCGGT	155
Rorα	XM_217192	CCCGATGTCTTCAAATCCTTAGG	TCAGTCAGATGCATAGAACACAAACTC	89
Ror γ	XM_006232926.3	GCAGGAGCAATGGAAGTCG	CGCTGAGGAAGTGGGAAAA	163
Rev-Erbα	NM_145775	ACAGCTGACACCACCCAGATC	CATGGGCATAGGTGAAGATTTCT	101
Nfil3	NM_053727.2	TCCGCTGGCAACCTTGGATA	CAGGGATGCAGGCTCCTTTT	224
tPA	XM_008771348.1	GTCAGATTCCAGTCAGTGTG	GTTGCTCGTGATGGTTTTG	229
Pai-1	NM_012620.1	GACAATGGAAGAGCAACATG	ACCTCGATCTTGACCTTTTG	205
uPA	NM_013085.3	TCGGACAAGAGAGTGCCA	TCACAATCCCGCTCAGAG	231
Plg	NM_053491.2	TGCGGAATCAGCCATAGTCC	GGGTTTGTCTGTGGGGTGAA	183

Table 2. Litter size and chronodisruption effect on birth body weight.

Protocol	LD	CPS
Liter size (number)	12,4±1,0	14,0±1,4
Newborn body weight (g)	6,66±0,06	6,93±0,07**
Male newborn body weight (g)	6,78±0,07	7,10±0,09**
Female newborn body weight (g)	6,51±0,1	6,77±0,09

LD: mothers under light-dark cycle 12/12. CPS: mothers under chronic photoperiod shift throughout gestation. The results are represented as mean±SEM with n=12 mother for LD and n=6 mother for CPS. **: Different from LD (P<0.01, unpaired t-test)

Supplemental Figure 1

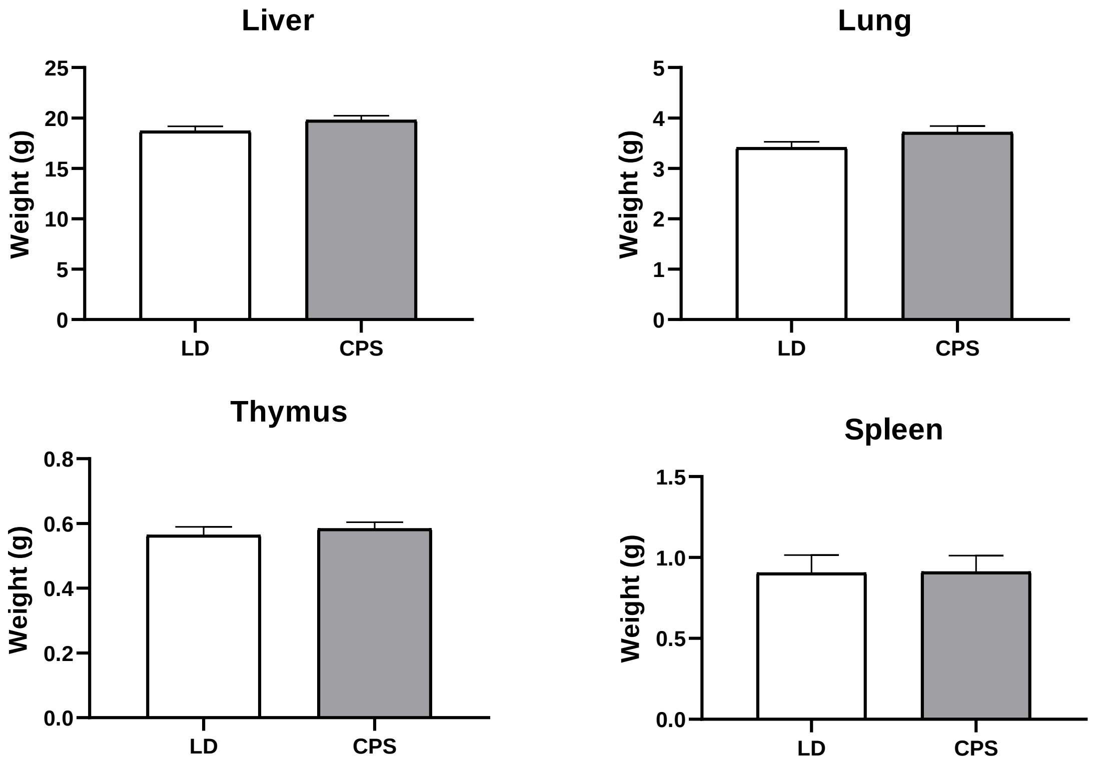

Figure 1. Weight of the organs of the adult progeny (P90) gestated under gestational chronodisruption. The data is represented as mean ± SEM. The white bar represents males gestated in LD and the gray bar represents males gestated in CPS. LD and CPS (n = 30) offspring. No statistically significant differences are observed; Liver P=0.11, Spleen P=0.78, Lung P=0.09 and Thymus P=0.54 (unpaired t-test).

Table 3. Analysis by single cosinor method for the transcriptional level of clock genes and plasminogen activator system genes in LD and CPS liver from male rats at 90-day-old.

Results for Protocol-Effects by Analysis Single Cosinor							
Gene	Protocol	p-value	q-value	R^2	A	M	ϕ (ZT)
Bmal1	LD	2,4E-13	3,6E-12	0,87	0,5	0,5	1,2
	CPS	9,24E-06	1,98E-05	0,55	0,2	0,2	1,5
Clock	LD	3,96E-04	8,49E-04	0,42	0,3	0,8	3,7
	CPS	3,72E-02	5,07E-02	0,20	0,3	0,9	0,4
Per 1	LD	1,32E-03	2,20E-03	0,37	3,1	2,6	14,0
	CPS	4,99E-05	8,73E-05	0,49	2,2	2,6	15,2
Per 2	LD	4,80E-06	1,80E-05	0,57	1,1	1,6	16,9
	CPS	5,24E-05	8,73E-05	0,49	1,2	1,2	15,6
Per 3	LD	2,50E-04	6,25E-04	0,44	57,4	37,8	14,0
	CPS	9,10E-09	6,83E-08	0,72	39,8	29,5	13,6
Cry1	LD	2,25E-03	3,38E-03	0,34	0,6	0,8	19,9
	CPS	2,79E-13	4,19E-12	0,86	0,6	0,8	19,3
Cry2	LD	1,39E-04	4,17E-04	0,46	0,6	1,5	16,4
	CPS	1,67E-06	4,18E-06	0,60	0,5	1,2	16,6
Rorα	LD	7,98E-01	7,98E-01	n.s.			
	CPS	1,50E-06	4,18E-06	0,60	0,59	1,12	21,2
Rorγ	LD	8,45E-10	6,34E-09	0,76	0,75	0,92	20,5
	CPS	2,30E-07	1,15E-06	0,65	1,34	1,61	19,8
Rev-Erbα	LD	1,77E-07	8,85E-07	0,66	35,61	25,19	10,4
	CPS	5,13E-07	1,92E-06	0,63	43,36	26,95	9,3
Nfil3	LD	1,29E-03	2,20E-03	0,37	0,29	0,64	0,7
	CPS	9,98E-04	1,50E-03	0,38	0,51	1,13	21,1
Pai-1	LD	1,96E-01	2,67E-01	n.s.			
	CPS	7,29E-01	7,29E-01	n.s.			
tPA	LD	2,86E-01	3,58E-01	n.s.			
	CPS	6,68E-02	8,35E-02	n.s.			
uPA	LD	7,97E-01	7,98E-01	n.s.			
	CPS	4,16E-01	4,46E-01	n.s.			
Plg	LD	7,90E-01	7,98E-01	n.s.			
	CPS	2,84E-01	3,28E-01	n.s.			

Parameters are p-value, rhythm detection considered statistically significant ($p < 0.05$); R^2, degree of adjustment of the curve to the data; A, amplitude; M, MESOR.
n.s., not significant, absence of daily rhythm.

Table 4. Analysis by JTK_Cycle method for the transcriptional level of clock genes and plasminogen activator system genes in LD and CPS liver from male rats at 90-day-old.

Results for Protocol-Effects by Analysis JTK_Cycle						
Gene	Protocol	p-values	BH Q	PER	LAG (ZT)	AMP
Bmal1	LD	3,58699E-10	1,34512E-09	24,00	2,0	0,4
	CPS	2,649E-09	7,94699E-09	20,00	4,0	0,1
Clock	LD	1,31E-03	2,19E-03	24,00	4,0	0,3
	CPS	1,43E-03	1,96E-03	24,00	0,0	0,3
Per 1	LD	3,25186E-07	9,75559E-07	20,00	14,0	1,5
	CPS	1,61986E-05	2,69977E-05	20,00	16,0	1,8
Per 2	LD	6,15478E-05	1,32E-04	24,00	16,0	1,0
	CPS	1,73186E-06	3,71112E-06	24,00	16,0	0,8
Per 3	LD	7,10828E-13	1,06624E-11	24,00	14,0	13,6
	CPS	4,33398E-10	2,16699E-09	24,00	14,0	20,6
Cry1	LD	2,03984E-05	5,09961E-05	24,00	22,0	0,5
	CPS	2,84E-10	2,13E-09	24,00	20,0	0,5
Cry2	LD	1,04E-04	1,96E-04	24,00	16,0	0,5
	CPS	7,94705E-06	1,49007E-05	24,00	16,0	0,5
Rorα	LD	1,00E+00	1,00E+00	n.s.		
	CPS	1,01E-06	2,52E-06	24,00	21,0	0,49
Rorγ	LD	1,80E-11	1,35E-10	24,00	20,0	0,57
	CPS	8,19E-10	3,07E-09	24,00	20,0	1,07
Rev-Erbα	LD	6,13E-11	3,07E-10	24,00	10,0	9,87
	CPS	2,82E-14	4,23E-13	24,00	10,0	9,30
Nfil3	LD	2,78E-03	4,17E-03	20,00	0,0	0,19
	CPS	1,10E-03	1,66E-03	24,00	20,0	0,46
Pai-1	LD	9,35E-01	1,00E+00	n.s.		
	CPS	8,29E-01	9,26E-01	n.s.		
tPA	LD	6,47E-01	8,83E-01	n.s.		
	CPS	1,25E-01	1,57E-01	n.s.		
uPA	LD	1,00E+00	1,00E+00	n.s.		
	CPS	9,35E-01	9,35E-01	n.s.		
Plg	LD	1,00E+00	1,00E+00	n.s.		
	CPS	8,64E-01	9,26E-01	n.s.		

Rhythm detection considered statistically significant ($p < 0.05$).

n.s., not significant, absence of daily rhythm.

Table 5. Analysis by RAIN's longitudinal mode method for the transcriptional level of clock genes and plasminogen activator system genes in LD and CPS liver from male rats at 90-day-old.

Results for Protocol-Effects by Analysis RAIN						
Gene	Protocol	p Val	q-value	phase (ZT)	peak.shape	period
Bmal1	LD	9,93E-16	1,49E-14	21,0	12,0	24,0
	CPS	2,99E-09	7,48E-09	1,0	8,0	24,0
Clock	LD	2,91E-04	4,37E-04	1,0	8,0	24,0
	CPS	1,29E-05	1,76E-05	21,0	12,0	24,0
Per 1	LD	1,65E-10	4,95E-10	9,0	16,0	24,0
	CPS	3,65E-07	5,48E-07	13,0	12,0	24,0
Per 2	LD	1,74E-06	3,73E-06	9,0	20,0	24,0
	CPS	5,66E-08	1,21E-07	13,0	12,0	24,0
Per 3	LD	1,15E-12	4,31E-12	9,0	12,0	24,0
	CPS	1,78E-10	6,68E-10	9,0	12,0	24,0
Cry1	LD	8,60E-08	2,15E-07	17,0	12,0	24,0
	CPS	2,06E-14	1,55E-13	13,0	16,0	24,0
Cry2	LD	2,03E-05	3,38E-05	13,0	12,0	24,0
	CPS	1,23E-07	2,05E-07	13,0	12,0	24,0
Rorα	LD	5,01E-01	5,46E-01	n.s.		
	CPS	4,56E-10	1,37E-09	17,0	12,0	24,0
Rorγ	LD	2,35E-13	1,76E-12	17,0	12,0	24,0
	CPS	2,72E-11	1,36E-10	17,0	12,0	24,0
Rev-Erbα	LD	1,10E-12	4,31E-12	5,0	12,0	24,0
	CPS	7,27E-19	1,09E-17	5,0	12,0	24,0
Nfil3	LD	5,64E-06	1,06E-05	21,0	8,0	24,0
	CPS	6,47E-08	1,21E-07	0,0	12,0	24,0
Pai-1	LD	3,37E-01	4,21E-01	n.s.		
	CPS	7,99E-02	9,22E-02	n.s.		
tPA	LD	9,71E-02	1,32E-01	n.s.		
	CPS	9,21E-03	1,15E-02	21,0	12,0	24,0
uPA	LD	8,89E-01	8,89E-01	n.s.		
	CPS	6,39E-01	6,39E-01	n.s.		
Plg	LD	5,10E-01	5,46E-01	n.s.		
	CPS	3,55E-01	3,80E-01	n.s.		

Rhythm detection considered statistically significant ($p < 0.05$).

n.s., not significant, absence of daily rhythm.

Supplemental Figure 2

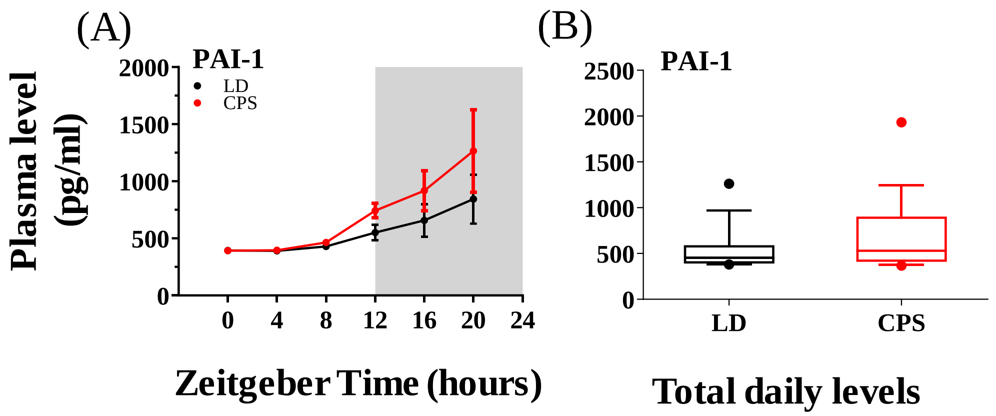

Figure 2. Preliminary quantification of daily plasma concentration of PAI-1 protein in 90-day-old male rats by ELISA. Plasma concentration of PAI-1 protein was assessed using an established ELISA method (PAI-1 (SERPINE1) Rat SimpleStep ELISATM Kit, catalog number ab201283; Abcam®) following the manufacturer's instructions. Data and median with interquartile range are shown, in LD and CPS adult offspring from each pregnancy condition (n=18, LD and CPS from 3 mothers each one) were anesthetized and blood samples were obtained every 4 hours for 6 samplings in a period of 20 hours, (n=3/each time point, for LD and CPS). **A) Detection of daily rhythm of PAI-1 plasma protein, (n=3/for each time point for LD and CPS) are shown.** Black symbols represent males gestated under control conditions (LD; black dots), and red symbols indicate males gestated in CPS (CPS; red dots). Time is expressed as zeitgeber time (ZT), with ZT0 as time lighting onset and ZT12 as lighting end; the gray bar indicates lights off. The RAIN's longitudinal mode, JTK_Cycle and the single cosinor method were used to determine daily rhythm ($p<0.05$), solid black and red lines represent the detection of a 24-hour daily rhythm for the three methods. No significant differences were found between CPS and LD for that time point (Mann-Whitney U test). **B) Daily profile of PAI-1 (n=18, LD and CPS)**. No significant differences were found between CPS and LD (Mann-Whitney U test).

As stated in the results section, daily rhythms were found for PAI-1 plasma protein concentration in both CPS and LD progeny; displaying an acrophase (ZT of the oscillation peak) in the active phase (dark) of the circadian cycle, in agreement with data previously reported by others in rodents (Ohkura et al., 2006). Despite finding no differences between CPS and LD groups in the plasma daily concentration of PAI-1, the amplitude of the oscillation of plasma concentration in the active phase was increased in adult males which had been gestated under CPS (A = 198,8 (LD) and 387,9 (CPS); AMP = 96,0 (LD) and 206,0 (CPS) determined by Cosinor and JTK_Cycle respectively) relative to LD adult offspring (see Table 6 below).

Table 6. Analysis by single cosinor, JTK_Cycle and RAIN method for plasma levels of PAI-1 in LD and CPS from male rats at 90-day-old.

Results for Protocol-Effects on PAI-1 plasma protein							
**Method**	**Protocol**	**p-value**	**q-value**	**R^2**	**A**	**M**	**ϕ ZT**
Cosinor	LD	1,53E-02	1,53E-02	0,39	198,8	544,3	18,7
	CPS	3,92E-03	7,85E-03	0,48	387,9	696,6	18,5
		**p-values**	**BH Q**	**PER**	**LAG(ZT)**	**AMP**	
JTK_Cycle	LD	1,06E-02	5,11E-03	24,00	18,0	96,0	
	CPS	2,55E-03	5,11E-03	24,00	16,0	206,0	
		**pVal**	**q-value**	**phase (ZT)**	**peak.shape**	**period**	
RAIN	LD	6,22E-04	6,22E-04	17,00	4,0	24,0	
	CPS	2,99E-06	5,98E-06	17,00	4,0	24,0	

Supplemental Figure 3

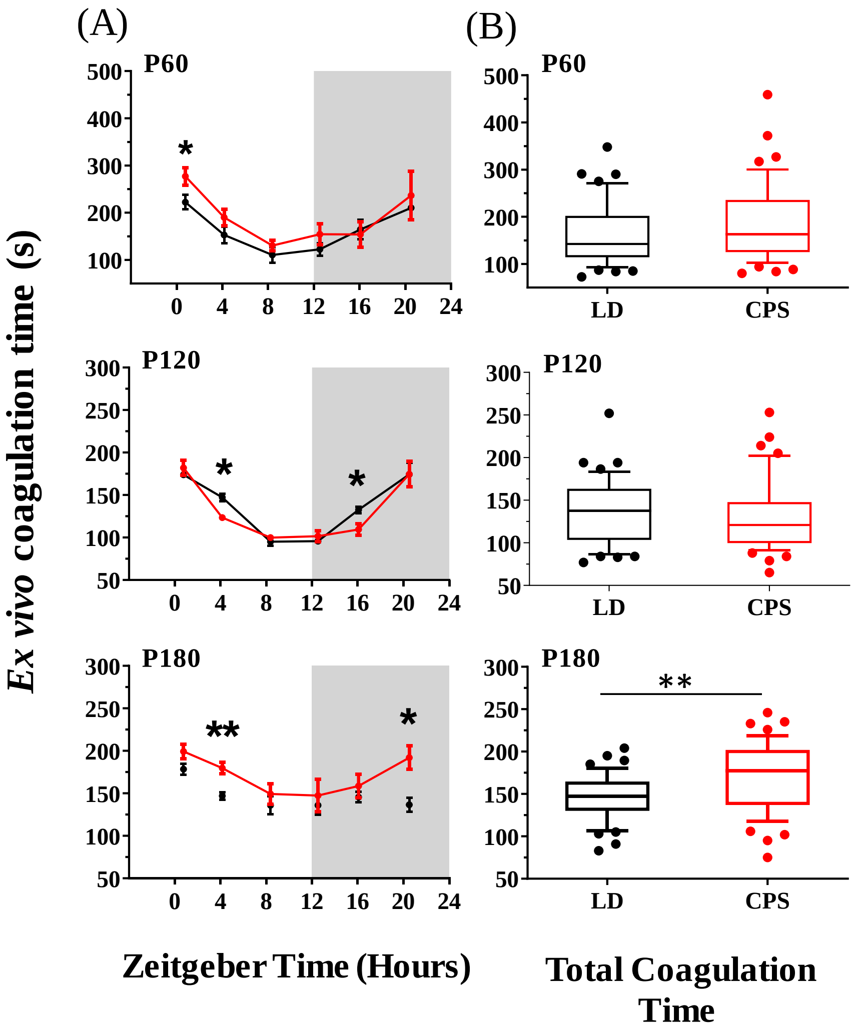

Figure 3. Effects of CPS on coagulation time ex vivo at postnatal age of 60, 120 and 180 days. Coagulation times in LD and CPS males (n=8/mothers for both groups) were measured every 4 hours for six samplings over 20 hours (n=8/each time point) starting at ZT1 and ending at ZT21, using the method described below. **A) Detection of daily rhythm of coagulation time at postnatal age of P60, P120 and P180 days are shown.** Black symbols represent males gestated under control conditions (LD; black dots), and red symbols indicate males gestated in CPS (CPS; red dots). Time is expressed as zeitgeber time (ZT), with ZT0 as time lighting onset and ZT12 as lighting end; the gray bar indicates lights off. The RAIN's longitudinal mode, JTK_Cycle and the single cosinor method were used to determine daily rhythm ($p<0.05$), solid black and red lines represent the detection of a 24-hour daily rhythm for the three methods. *$p<0.05$, **$p<0.01$ Different from LD for time point (Unpaired t-test). **B) Daily total coagulation time for each respective postnatal age (n=48 males for LD and CPS)**. **$p<0.01$ Different from LD (Unpaired t-test).

Table 7. Analysis by Single Cosinor method for coagulation time in LD and CPS male rats.

Results for Protocol-Effects on Coagulation Time by Analysis Single Cosinor							
Age	Protocol	p-value	q-value	R^2	A	M	ϕ ZT
P60	LD	4,23E-03	6,35E-03	0,21	41,2	163,9	23,8
	CPS	4,37E-04	6,56E-04	0,28	61,1	185,7	0,2
P120	LD	3,50E-15	1,05E-14	0,76	45,6	136,5	23,4
	CPS	1,09E-10	3,27E-10	0,62	45,1	131,8	23,5
P180	LD	2,21E-02	2,21E-02	0,15	14,6	146,6	1,1
	CPS	1,02E-03	1,02E-03	0,25	28,7	171,1	0,2

Table 8. Analysis by JTK_Cycle method for coagulation time in LD and CPS male rats.

Results for Protocol-Effects on Coagulation Time by Analysis JTK_Cycle						
Age	Protocol	p-values	BH Q	PER	LAG (ZT)	AMP
P60	LD	2,05E-02	3,07E-02	24,00	0,0	31,5
	CPS	3,30E-12	9,89E-12	24,00	0,0	32,5
P120	LD	1,85E-13	5,56E-13	24,00	0,0	42,4
	CPS	8,37E-04	1,03E-03	24,00	2,0	50,9
P180	LD	9,92E-02	9,92E-02	n.s		
	CPS	1,03E-03	1,03E-03	24,00	0,0	29,3

Table 9. Analysis by RAIN method for coagulation time in LD and CPS male rats.

Results for Protocol-Effects on Coagulation Time by Analysis RAIN						
Age	Protocol	pVal	q-value	phase (ZT)	peak.shape	period
P60	LD	9,11E-04	1,37E-03	21,0	12,0	24,0
	CPS	1,26E-05	2,53E-05	21,0	8,0	24,0
P120	LD	4,95E-16	1,48E-15	21,0	8,0	24,0
	CPS	5,43E-13	1,63E-12	21,0	8,0	24,0
P180	LD	6,39E-03	6,39E-03	21,0	12,0	24,0
	CPS	2,91E-04	2,91E-04	21,0	12,0	24,0

Ex vixo blood coagulation method

Blood clotting time was measured as reported previously ElGendy and Abbas (ElGendy and Abbas, 2014). Briefly, male rats were deeply anesthetized (isoflurane 3.5%, Baxter Laboratories), the tail of the animal was warmed for 1 min in the water at 40 °C. The tail was dried and cut at the tip with a razor blade. A 25-μL sample of capillary blood was collected into a microhematocrite glass capillary. The chronometer was started when the blood first made contact with the tube. The blood was left to flow by gravity between the two marks of the tube, 45 mm apart, by tilting the capillary tube alternatively to +60° and −60° angles with respect to the horizontal plane until blood ceased to flow (reaction endpoint).

Reference

ElGendy, A. A., and Abbas, A. M. (2014). Effects of warfarin and l-carnitine on hemostatic function and oxidative stress in streptozotocin-induced diabetic rats. *J. Physiol. Biochem.* 70, 535–546. doi:10.1007/s13105-014-0333-4.

Ohkura, N., Oishi, K., Fukushima, N., Kasamatsu, M., Atsumi, G. I., Ishida, N., et al. (2006). Circadian clock molecules CLOCK and CRYs modulate fibrinolytic activity by regulating the PAI-1 gene expression. *J. Thromb. Haemost.* 4, 2478–2485. doi:10.1111/j.1538-7836.2006.02210.x.
